# Neurofilament light chain plasma concentration predicts neurodegeneration and clinical progression in nondemented elderly adults

**DOI:** 10.18632/aging.102220

**Published:** 2019-09-12

**Authors:** Hao Hu, Ke-Liang Chen, Ya-Nan Ou, Xi-Peng Cao, Shi-Dong Chen, Mei Cui, Qiang Dong, Lan Tan, Jin-Tai Yu, Alzheimer’s Disease Neuroimaging Initiative

**Affiliations:** 1 Department of Neurology, Qingdao Municipal Hospital, Qingdao University, Qingdao, China; 2 Department of Neurology and Institute of Neurology, WHO Collaborating Center for Research and Training in Neurosciences, Huashan Hospital, Shanghai Medical College, Fudan University, Shanghai, China; 3 Clinical Research Center, Qingdao Municipal Hospital, Qingdao University, Qingdao, China

**Keywords:** neurofilament light chain, Alzheimer’s disease, biomarker, prediction

## Abstract

Previous studies demonstrated that plasma neurofilament light chain (NFL) played important predictive roles in disease progression and neurodegeneration in the preclinical phase of familial Alzheimer’s disease (AD). However, whether plasma NFL has the same predictive roles in sporadic AD is still unclear. In this study, 243 cognitively normal (CN) participants from the ADNI database were divided into two subgroups (CN- and CN+) according to CSF Aβ or AV45-PET. Associations of baseline plasma NFL concentrations or rate of change in plasma NFL with longitudinal data on other biomarkers were tested by multivariate linear mixed effects models (LMEMs). Results showed that plasma NFL concentration and its rate of change were already abnormally high in the preclinical phase of AD. Plasma NFL was associated with three core AD-related biomarkers in preclinical phase. Baseline plasma NFL, but not its rate of change, played predictive roles in both cognitive decline (β = -0.0349, p = 0.0274) and hippocampal atrophy (β = -0.0351, p = 0.0088), especially for preclinical AD participants. In summary, these results indicated that baseline plasma NFL, but not its rate of change, may be a valuable noninvasive tool to assess neurodegeneration and predict longitudinal disease progression in preclinical AD individuals.

## INTRODUCTION

Available evidence strongly supports the notion that the initiating event in Alzheimer’s disease (AD) is related to abnormality of β-amyloid (Aβ) peptide, which occurs when individuals are still cognitively normal [1–5].

Based on this notion, current studies have divided the clinical stages of AD into three phases: preclinical phase, prodromal phase and dementia phase [6]. Among these phases, the most critical stage for early diagnosis and monitoring is the preclinical phase in which individuals are cognitively normal but have had pathological changes. These pathological changes can be reflected by some biomarkers, such as cerebrospinal fluid Aβ (CSF Aβ), cerebrospinal fluid total tau protein (CSF t-tau), cerebrospinal fluid phosphorylated tau protein (CSF p-tau), F18-fluorodeoxyglucose positron emission tomography (FDG-PET) and magnetic resonance imaging (MRI). However, none of these biomarkers is suited to track disease progression, because of their invasiveness or high expenses. Therefore, many studies focused on blood biomarkers, such as plasma neurofilament light (NFL).

The NFL is a component of the axonal cytoskeleton and a putative marker of large-caliber axonal degeneration which is an important pathological change in neurodegeneration diseases [7]. Advancements in measurements of NFL have revealed the abnormal increase in the progression of AD, even in early stages. As for familial AD, a recent study has found important predictive roles of serous NFL in disease progression and brain neurodegeneration at the preclinical phase [8]. However, whether blood NFL has the same predictive roles in sporadic AD is still unclear. Another recent study focusing on cognitively normal (CN) individuals has found that elevated levels of CSF NFL but not CSF tTau, pTau or neurogranin (Ng) were a risk factor for mild cognitive impairment (MCI) [9]. Moreover, advancements in measurements of NFL have revealed strong correlations between CSF NFL and blood NFL, which has sparked interests in roles of blood NFL on sporadic AD [10]. Therefore, we speculated that, as for sporadic AD, plasma NFL also had some predictive roles in neurodegeneration or progression of disease in the preclinical phase. This study focused on CN individuals to test the hypothesis that the plasma NFL concentration and its rate of change are abnormally elevated in the preclinical phase of AD and they correlate with impaired cognition, neuroimaging abnormalities and CSF biomarkers of AD.

## RESULTS

### Sample characteristics

**Figure 1:**
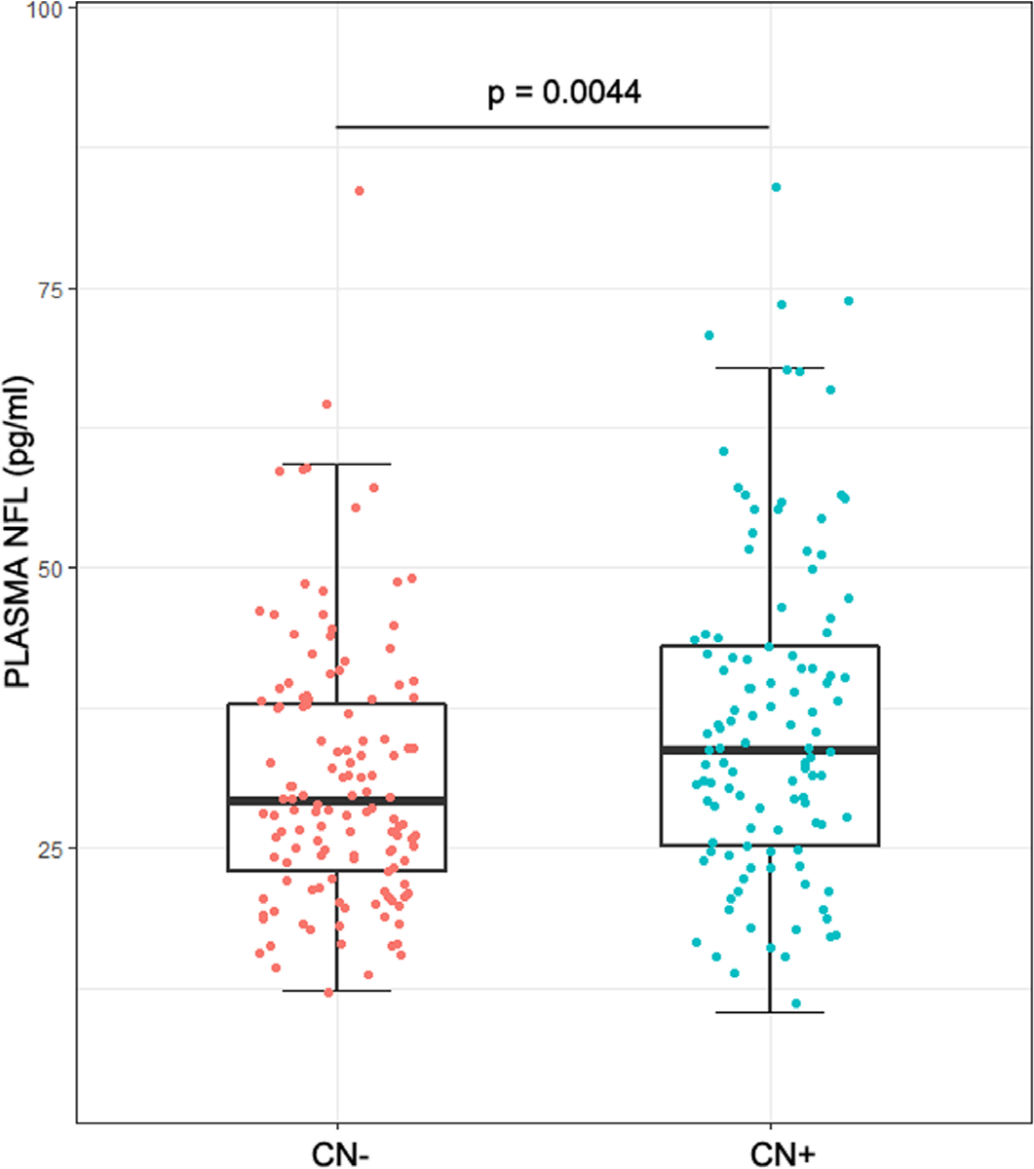
**Plasma NFL in different groups.** The intergroup differences in plasma NFL were tested using Wilcoxon test. Plasma NFL concentrations were higher in the CN+ group with a high degree of overlap between the two groups (p = 0.0044).

The demographics of the study population were listed in Table 1. No statistical difference was found between CN- group (healthy control group: cognitively normal participants without significant Aβ-related pathological changes) and CN+ group (preclinical group: cognitively normal participants with significant Aβ-related pathological changes) in age (p = 0.0510), sex (p = 0.0679) and education (p = 0.1005). The prevalence of an APOEε4 in CN+ group was higher than that in CN- group. Plasma NFL correlated with age (ρ = 0.47, p < 0.01), but not with sex (median, 30.9 pg/ml for male vs 31.7 pg/ml for female, p = 0.44), education (ρ = 0.01, p = 0.87) or APOEε4 genotype (median, 30.6 pg/ml for carriers vs 31.8 pg/ml for noncarriers, p = 0.60). As for CSF biomarkers, the levels of Aβ42, tTau and p-tau181 were much higher in CN+ group (Aβ42: p < 0.0001; t-tau: p = 0.0002; p-tau181: p < 0.0001) compared with the CN- group. However, the cognitive level (ADNI_MEM: p = 0.1052), FDG-PET (p = 0.1525) and hippocampus volume (p = 0.0734) showed no significant differences between the two groups. It is worth noting that both plasma NFL concentration and its rate of change were higher in the CN+ group (NFL concentration: p = 0.0044; Rate of change: p < 0.0001) (Table 1, Figure 1). However, the two groups showed a high degree of overlap in the range of plasma NFL concentration as well as that of its change rate (Table 1, Figure 1).

**Table 1:** Demographics for CN population.

**Variable**	**CN-**	**CN+**	**Total**	**p value**
**N**	130	113	243	-
**Age, Mean (SD), years**	72.77 (5.54)	73.33 (6.21)	72.76 (6.80)	0.0510
**Female, N (%)**	55 (42.31)	62 (54.87)	117 (48.15)	0.0679
**Education, Mean (SD), years**	16.85 (2.64)	16.35 (2.54)	16.62 (2.60)	0.1005
**APOEε4, %**	13.85	43.36	27.57	<0.0001^a^
**ADNI_MEM, Mean (SD)**	1.13 (0.58)	1.0 (0.59)	1.07 (0.59)	0.1052
**ADNI_EF, Mean (SD)**	0.95 (0.79)	0.65 (0.80)	0.81 (0.81)	0.0031^a^
**CSF Aβ42, Mean (SD), pg/ml**	237.98 (25.62)	154.732 (32.47)	199.27 (50.68)	<0.0001^a^
**CSF t-tau, Mean (SD), pg/ml**	57.06 (22.67)	77.47 (39.93)	66.55 (33.41)	0.0002^a^
**CSF p-tau181, Mean (SD), pg/ml**	26.95 (10.90)	41.65 (24.66)	33.79 (19.97)	<0.0001^a^
**Hippocampus Volume, Mean (SD), mm^3^**	7618.49 (741.95)	7440.13 (821.38)	7535.55 (783.34)	0.0734
**FDG-PET**	1.32. (0.11)	1.30 (0.12)	1.32 (0.11)	0.1525
**Plasma NFL, Mean (SD), pg/ml**	31.61 (13.10)	36.39 (14.46)	33.84 (13.93)	0.0044^a^
**Rate of change Plasma NFL, year**	2.34	2.94	-	<0.0001^a^
**Mean (CI), pg/ml**	(1.47~3.25)	(1.83~4.04)	-	-

Abbreviations: APOE, apolipoprotein E; ADNI, Alzheimer’s Disease Neuroimaging Initiative; ADNI_MEM, memory domain composite score; ADNI_EF, executive domain composite score; CSF, cerebrospinal fluid; Aβ, amyloid-β; t-tau, total tau protein; p-tau, phosphorylated tau protein; NFL, neurofilament light chain; SD, standard deviation; CI, confidence interval; CN-, cognitively normal participants with negative Aβ; CN+, cognitively normal participants with positive Aβ

^a^ Lower-case letter indicated that the results were statistically significant.

### Cross-sectional associations of plasma NFL with CSF biomarkers, cognition and hippocampus volume

Table 2 showed the results of a multiple linear regression analysis for cross-sectional associations. Among total CN participants, a significant association was found between CSF Aβ42 and plasma NFL concentration (β = -0.1421, p = 0.0320), which was not found in the two subgroups. Moreover, significant associations between CSF t-tau and plasma NFL concentration were found in both total CN participants (β = 0.1821, p = 0.0091) and CN+ subgroup (β = 0.29319, p = 0.0042). There was no significant association of plasma NFL concentration with CSF p-tau181, FDG-PET, ADNI_MEM, ADNI_EF, and hippocampus volume.

**Table 2:** Cross-sectional associations of plasma NFL with CSF biomarkers, cognition and hippocampus volume.

**Variable**	**CN-**			**CN+**			**All participants**	
	**β**	**p value**		**β**	**p value**		**β**	**p value**
**CSF Aβ42**	-0.0215	0.827		0.02457	0.8109		-0.1421	0.0320a
**CSF t-tau**	-0.0325	0.741		0.29319	0.0042a		0.1821	0.0091a
**CSF p-tau181**	-0.0353	0.717		0.095684	0.3604		0.0887	0.2011
**FDG-PET**	-0.0454	0.6365		-0.09396	0.3821		-0.0811	0.2583
**ADNI_MEM**	0.0103	0.8375		0.024174	0.6714		0.0098	0.7946
**ADNI_EF**	0.0634	0.3728		-0.0243	0.7572		-0.0047	0.9311
**Hippocampus Volume**	-0.0797	0.3178		-0.0336	0.7360		-0.0752	0.2290

Abbreviations: ADNI, Alzheimer’s Disease Neuroimaging Initiative; ADNI_MEM, memory domain composite score; ADNI_EF, executive domain composite score; CSF, cerebrospinal fluid; Aβ, amyloid-β; t-tau, total tau protein; p-tau, phosphorylated tau protein; FDG-PET, F18-fluorodeoxyglucose positron emission tomography; CN-, cognitively normal participants with negative Aβ; CN+, cognitively normal participants with positive Aβ

^a^ Lower-case letter indicated that the results were statistically significant.

Cross-sectional associations between plasma NFL concentrations and other biomarkers at baseline were tested by multiple linear regression models corrected for age, sex, education and APOE genotype.

### Associations between rate of change in plasma NFL and rates of change in some other biomarkers

Associations between estimated annual rate of change in plasma NFL and estimated annual rates of change in some other biomarkers for CN- participants and CN+ participants were shown in Table 3. Results from multivariate linear mixed effects models (LMEMs) revealed significant associations between rate of change in CSF Aβ42 and rate of change in plasma NFL in CN+ participants (β = -3.2697, p = 0.0027) and total CN participants (β = -2.1670, p = 0.0059) but not in CN- participants (β = -1.4466, p = 0.2364). Although results also showed the associations between rate of change in plasma NFL and rates of change in FDG-PET (β = -0.0047, p = 0.0451) and ADNI_MEM (β = -0.0177, p = 0.0482) in total CN participants, these associations were not found in the two subgroups.

**Table 3:** Associations between rate of change in plasma NFL and rates of change in other biomarkers.

**Variable**	**CN-**			**CN+**			**All participants**	
	**β**	**p value**		**β**	**p value**		**β**	**p value**
**CSF Aβ42**	-1.4466	0.2364		-3.2697	0.0027^a^		-2.1670	0.0059^a^
**CSF t-tau**	0.9519	0.2780		1.1780	0.3870		1.2526	0.1100
**CSF p-tau181**	-0.5848	0.4607		1.7722	0.2374		0.7792	0.3500
**FDG-PET**	-0.0074	0.0501		-0.0016	0.6448		-0.0047	0.0451^a^
**ADNI_MEM**	-0.0040	0.6923		-0.0317	0.0573		-0.0177	0.0482^a^
**ADNI_EF**	-0.0157	0.2706		-0.0292	0.1804		-0.0067	0.5945
**Hippocampus Volume**	0.0037	0.6641		-0.0249	0.0862		-0.0089	0.2780

Abbreviations: ADNI, Alzheimer’s Disease Neuroimaging Initiative; ADNI_MEM, memory domain composite score; ADNI_EF, executive domain composite score; CSF, cerebrospinal fluid; Aβ, amyloid-β; t-tau, total tau protein; p-tau, phosphorylated tau protein; FDG-PET, F18-fluorodeoxyglucose positron emission tomography; CN-, cognitively normal participants with negative Aβ; CN+, cognitively normal participants with positive Aβ

^a^ Lower-case letter indicated that the results were statistically significant.

The rate rate of change in plasma NFL was was extracted from LMEMs. Associations between rate of change in plasma NFL and longitudinal data of other biomarkers (CSF Aβ42, CSF t-tau, CSF p-tau181, FDG-PET, ADNI_MEM, ADNI_EF, Hippocampus Volume) were tested by LMEMs corrected for age, sex, education and APOE genotype.

### Prediction of changes in hippocampus volume and cognition by baseline plasma NFL and rate of change in plasma NFL

**Figure 2:**
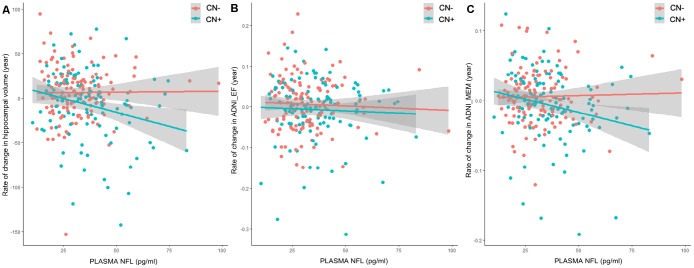
**Prediction of changes in hippocampus volume and cognition by baseline plasma NFL.** Associations between baseline plasma NFL concentrations or rate of change in plasma NFL and longitudinal data were tested by LMEMs corrected for age, sex, education and APOE genotype. (**A**) In CN+ group, higher baseline plasma NFL levels were significantly associated with an increased rate of change in hippocampus volume (β = -0.0351, p = 0.0088). This association did not exist in CN- group (β = -0.0007, p = 0.9311). (**B**) Results did not show association between baseline plasma NFL levels and ADNI_EF (CN-: β = -0.0076, p = 0.5975; CN+: β = -0.0135, p = 0.5070). (**C**) In CN+ group, higher baseline plasma NFL levels were significantly associated with an increased rate of change in ADNI_MEM (β = -0.0349, p = 0.0274). This association did not exist in CN- group (β = -0.0009, p = 0.9313).

In CN+ group, a higher baseline plasma NFL level was significantly associated with an increased rate of change in both ADNI_MEM (β = -0.0349, p = 0.0274) and hippocampus volume (β = -0.0351, p = 0.0088), but not in ADNI_EF (β = -0.0135, p = 0.5070) (Table 4, Figure 2). Moreover, higher CSF Aβ42 and p-tau181 levels were significantly associated with an increased rate of change in ADNI_MEM (β = -0.0429, p = 0.0120) and a higher CSF t-tau level was significantly associated with an increased rate of change in hippocampus volume (β = -0.0372, p = 0.0061) (Table 4). In CN- group, we did not find any association between these biomarkers (plasma NFL, CSF Aβ42, CSF t-tau, CSF p-tau181 and FDG-PET) and rate of change in cognition or hippocampus volume.

**Table 4:** Prediction of changes in hippocampus volume and cognition by biomarkers

	**Variable**	**CN-**			**CN+**	
		**β**	**p value**		**β**	**p value**
***ADNI_MEM***	**Plasma NFL**	-0.0009	0.9313		-0.0349	0.0274^a^
	**Rate of change (NFL)**	-0.0040	0.6923		-0.0317	0.0573
	**CSF Aβ42**	0.0162	0.1216		0.0387	0.0113^a^
	**CSF t-tau**	-0.0130	0.1818		-0.0305	0.0513
	**CSF p-tau181**	0.0111	0.2698		-0.0429	0.0120^a^
	**FDG-PET**	0.0112	0.2599		0.0225	0.1679
***ADNI_EF***	**Plasma NFL**	-0.0076	0.5975		-0.0135	0.5070
	**Rate of change (NFL)**	-0.0157	0.2706		-0.0292	0.1804
	**CSF Aβ42**	0.0116	0.4493		0.0021	0.9140
	**CSF t-tau**	0.0179	0.2305		-0.0322	0.1080
	**CSF p-tau181**	-0.0093	0.5289		-0.0231	0.2810
	**FDG-PET**	0.0031	0.8334		0.0171	0.4095
***Hippocampus***	**Plasma NFL**	-0.0007	0.9311		-0.0351	0.0088^a^
***Volume***	**Rate of change (NFL)**	0.0037	0.66405		-0.0249	0.0862
	**CSF Aβ42**	0.0001	0.9950		0.0209	0.1360
	**CSF t-tau**	-0.0040	0.6370		-0.0372	0.0061^a^
	**CSF p-tau181**	0.0084	0.3380		-0.0196	0.1713
	**FDG-PET**	0.01008	0.25348		0.01605	0.2432

Abbreviations: ADNI, Alzheimer’s Disease Neuroimaging Initiative; ADNI_MEM, memory domain composite score; ADNI_EF, executive domain composite score; NFL, neurofilament light chain; CSF, cerebrospinal fluid; Aβ, amyloid-β; t-tau, total tau protein; p-tau, phosphorylated tau protein; FDG-PET, F18-fluorodeoxyglucose positron emission tomography; CN-, cognitively normal participants with negative Aβ; CN+, cognitively normal participants with positive Aβ

^a^ Lower-case letter indicated that the results were statistically significant.

Associations between baseline biomarkers (plasma NFL concentrations, rate of change in plasma NFL, CSF Aβ42, CSF t-tau, CSF p-tau181 and FDG-PET) and longitudinal data (ADNI_MEM, ADNI_EF and hippocampus volume) were tested by LMEMs corrected for age, sex, education and APOE genotype.

## DISCUSSION

Our study is the first to focus on cognitively normal individuals to explore the value of plasma NFL in the early prediction of sporadic AD. The main findings of our study were that (1) plasma NFL concentration and rate of change in plasma NFL were abnormally high in the preclinical phase of AD (2) plasma NFL was associated with some core AD-related biomarkers in the preclinical phase of AD (3) baseline plasma NFL played predictive roles in both impaired cognition and neuroimaging abnormalities, especially for the preclinical AD individuals (4) rate of change in plasma NFL showed predictive effects on neither of impaired cognition and neuroimaging abnormalities among CN individuals.

The higher level of baseline plasma NFL concentrations in CN+ group indicated that plasma NFL concentrations have already been abnormally high in the preclinical phase of AD. This result contradicted a previous study which did not find statistical differences in plasma NFL concentration between CN+ and CN- groups [10]. It is worth noting that our study included a larger cognitively normal cohort with more stringent quality control and excluded extremes (limit: mean±3SD). Moreover, to avoid misclassifying some preclinical AD patients in the category of the normal group, we divided participants into Aβ positive or negative (A+/A-) patients according CSF Aβ or AV45-PET SUVR. Therefore, data quality in our study was higher than that in the previous study. Three CSF biomarkers (Aβ42, t-tau, p-tau181) all significantly increased in CN+ group, whereas baseline cognitive level (especially ADNI_MEM), hippocampus volume and FDG-PET showed no obvious differences compared with the CN- group. These results were consistent with previous assumptions. A widely accepted assumption was that biomarkers have already been abnormal in the preclinical phase without significant cognitive decline. Among these biomarkers, some core biomarkers (especially Aβ and tau protein) became abnormal earlier than other markers (such as MRI and FDG-PET) [11]. However, though our results showed that plasma NFL concentration was already abnormally high in the preclinical AD phase, we could see that range of plasma NFL concentration in the two groups had considerable overlap, indicating that, unlike Aβ, plasma NFL may not be suitable as an early diagnostic marker.

Consistent with previous studies, our study found a cross-sectional association between plasma NFL and CSF t-tau. Previous studies showed that both CSF tau and plasma NFL, as indicators of neuronal injury, may reflect the degree of neurodegenerative changes [12–14]. It is worth noting that results also showed an association between plasma NFL and CSF Aβ42. An association between plasma NFL and CSF Aβ42 was found in total participants by the cross-sectional association test. Moreover, longitudinal results also showed associations between estimated annual rate of change in plasma NFL and estimated annual rate of change in CSF Aβ42 for total participants and CN+ participants, but not for CN- participants. These results contradicted previous studies which did not find associations between plasma NFL and CSF Aβ42 [10]. We thought the main reason for these contradictory results was that our study focused on the cognitively normal population. Previous studies indicated that Aβ-plaque accumulation in individuals destined to become dementia might begin two decades before the clinical symptoms [15]. Thus in the CN phase, not all individuals’ CSF Aβ42 had reached the plateau stage and the changes of CSF Aβ42 were still reflected by plasma NFL. According to the above results, we could see that plasma NFL was already abnormally high in the preclinical phase of AD and was associated with some early core AD-related biomarkers (CSF Aβ42 and tau protein).

In order to explore the utility of plasma NFL as a predictor of subsequent neurodegeneration and clinical symptoms and to compare predictive roles of plasma NFL with those of some other biomarkers, we used a (retrospective) pseudo-predictive analysis. The results showed that baseline plasma NFL could predict future annualized changes of hippocampal atrophy and annualized changes of ADNI_MEM especially for CN+ participants. Compared with plasma NFL, CSF p-tau181 played predictive roles only in cognitive decline for CN+ participants and CSF t-tau play predictive roles only in hippocampal atrophy for CN+ participants. According to previous studies, earliest cognitive symptoms in AD were characterized by typical deficits in episodic memory (ADNI-MEM) rather than executive functioning (ADNI-EF) [11]. Previous autopsy data confirmed that cerebral atrophy was one of the neurodegenerative biomarkers which indicated dendritic pruning as well as loss of synapses and neurons [16]. Some studies implied that cerebral atrophy (especially hippocampal volume) was the most proximate pathological substrate of cognitive impairment in AD [17–19]. Overall, these results indicated that, even in the preclinical phase of AD, plasma NFL still played predictive roles not only in cerebral structural changes but also in cognitive impairment. Therefore, though plasma NFL may not be suitable as an early diagnostic marker, it may be suitable as an early neurodegenerative biomarker in preclinical AD.

Blood-based biomarkers have obvious advantages in monitoring disease progression. Several previous studies have tested some blood-based biomarkers. However, there was heterogeneity in the results. As for plasma tau protein, a study found associations between increased plasma tau protein and AD hallmarks, but these associations were mild and they varied with cohorts [20]. Though a comprehensive meta-analysis of blood markers showed total tau protein was useful in differentiating participants with established AD from healthy controls yet with some overlaps, no evidence showed that it was useful in detection of earlier disease [21]. As for plasma Aβ, previous studies also failed to obtain consistent results to prove its ability to diagnose of AD [21]. Moreover, Aβ-related biomarkers may not track progression unless in very early stage of disease because Aβ-related pathology is an early event in AD and it may reach the plateau before symptom onset. Therefore, the above blood-based biomarkers seemed not to be suitable as early biomarkers in preclinical AD and our results may provide a new candidate.

A few limitations in our study should be noted. Firstly, we had some missing data in our longitudinal data, especially at later follow-up time points, which had a slight impact on the results. Secondly, the sample size in our study was comparatively small and these results should be further tested in a larger population. Thirdly, increased plasma NFL concentrations were also found in some other neurodegenerative diseases, such as frontotemporal dementia (FTD) and progressive supranuclear palsy (PSP), indicating a lack of specificity of plasma NFL [22, 23]. Though this limitation does not negate its role as a general biomarker for neurodegeneration, more studies involving a variety of neurodegenerative diseases are warranted to investigate the roles of plasma NFL.

Taken together, our findings suggested that plasma NFL concentrations and rate of change in plasma NFL were already abnormally high in the preclinical AD phase. Plasma NFL was associated with some core AD-related biomarkers, cognitive decline and cerebral atrophy, especially for the preclinical AD individuals. Therefore, plasma NFL may be a valuable noninvasive tool to assess neurodegeneration and to predict preclinical AD individuals’ longitudinal disease progression. However, the predictive roles of rate of change in plasma NFL were limited in the preclinical phase of sporadic AD.

## METHODS

### ADNI database

Data used in this study were obtained from the Alzheimer’s Disease Neuroimaging Initiative (ADNI) database (http://adni.loni.usc.edu). The ADNI database was launched in 2003 as a public-private partnership, led by the principal investigator Michael W. Weiner, MD. The ADNI participants have been recruited from more than 50 sites across the United States and Canada. The primary objective of the ADNI has been to test whether serial MRI, PET, other biological markers, and clinical or neuropsychological assessment can be combined to measure the progression of MCI and early AD. ADNI database consists of three parts, including the ADNI 1, the ADNI Grand Opportunities (GO) and the ADNI 2. To date, these 3 protocols have recruited more than 1500 adults (age range, 55–90 years) to participate in the research, including CN older individuals, persons with early or late MCI, and patients with early AD. The follow-up duration for each study group was specified in the protocols for the ADNI 1, ADNI 2, and ADNI GO. Regional ethics committees of all institutions approved of the study. Written informed consent was obtained from all study participants.

### Population

The study only included the CN participants. CN participants were defined as individuals with a Mini-Mental State Examination (MMSE) score from 25 to 30 inclusive, Clinical Dementia Rating (CDR) score of 0, no evidence of depression, and no memory complaints. In the ADNI database, the number of CN participants who have baseline plasma NFL samples is 482. We chose 245 participants who have available baseline CSF samples as well as neuroimaging and cognitive data. All the participants had at least one follow-up data of all biomarkers. The minimum follow-up time was one year and the maximum follow-up time was ten years. More than 75 percent of participants had at least three years of follow-up. Then we remove the extremums that were higher than three standard deviations (SD) above the mean or lower than three standard deviations below the mean (limited: mean±3SD). This step removed two extremes. Eventually, 243 CN participants were included in our study. According to the National Institute of Aging-Alzheimer’s Association (NIA-AA) research framework, Aβ-related biomarkers (CSF Aβ and Aβ-PET) were regarded as the core markers for the diagnosis of AD continuum [6]. In our study, Aβ positive or negative (A+/A-) participants were distinguished by a previously established cutoff of 192 pg/ml (CSF Aβ) or 1.11 (AV45-PET SUVR) [24]. Then CN participants were divided into two subgroups: participants with A- (CN-) and participants with A+ (CN+). We regarded the CN+ participants as patients with preclinical AD and regarded CN- participants as controls. The final groups are presented in Table 1.

### Cognitive assessments

Cognition was assessed by ADNI_EF and ADNI_MEM, which were two composite scales for executive functioning (ADNI_EF) and memory (ADNI_MEM) using data from the ADNI neuropsychological battery via item response theory methods. Compared with the separate scales, these two composite scales can reflect the cognitive level more robustly.

### Measurement of CSF biomarkers

Aβ42, t-tau, and p-tau181 were measured by the ADNI Biomarker Core (University of Pennsylvania) using the multiplex xMAP Luminex platform (Luminex Corp, Austin, TX, USA) with the INNOBIA AlzBio3 kit (Fujirebio, Ghent, Belgium) as described previously [1].

### Measurement of neuroimaging

MRI brain scans were acquired using 1.5-T imaging systems with a standardized protocol that included T1-weighted images using a sagittal, volumetric, magnetization-prepared rapid acquisition with gradient echo sequence. Automated volume measures were obtained with FreeSurfer (http://surfer.nmr.mgh.harvard. edu/fswiki). Our study used averaged volume measurements for the right and left hippocampi. The cerebral brain metabolism was tested by mean FDG-PET counts of lateral and medial frontal regions, anterior and posterior cingulate regions, as well as lateral parietal and temporal regions. The ADNI Florbetapir summary data (AV45-PET) were calculated using the cortical summary regions of interest (ROI) divided by the whole cerebellum reference region (SUMMARYSUVR_WHOLECEREBNORM).

### Measurement of plasma NFL concentrations

Blood samples were collected, processed, aliquoted, and frozen at -80°C according to standardized procedures. Plasma NFL concentrations were measured on an ultrasensitive single-molecule array platform with a home brew kit (Simoa Homebrew Assay Development Kit; Quanterix Corporation) using the same methodology as described previously [25]. The assay used a combination of monoclonal antibodies and purified bovine NFL as a calibrator. The relative error of the blank calculated concentrations was below 20% for all calibrators, run in triplicate, resulting in lower limits of quantifications of 2.2 pg/ml and upper limits of quantification of 1620 pg/ml. All samples measured within the range spanned by the limits of quantifications, and for the low quality control samples with a concentration of 14 pg/ml, the intra-assay coefficient of variation was 11.0% and intermediate precision was 11.1%. For the high-concentration quality control samples with a concentration of 137 pg/ml, the intra-assay coefficient of variation was 8.8% and intermediate precision was 9.6%. The measurements were performed in September 2016.

### Statistical analysis

The Mann-Whitney test and the Spearman rank correlation test were used to test the correlations between plasma NFL concentration and demographic factors including age, sex, apolipoprotein Eε4 genotype (APOEε4), and education. The intergroup differences in demographic data and biomarkers were tested using the Wilcoxon test. The distribution of plasma NFL data was non-normal and therefore normal transformation was performed prior to subsequent analysis (using z-scale). The rate of change in plasma NFL was modeled using a multivariate linear mixed effects model (LMEM) with fixed effects of time from baseline, diagnostic status, and interaction between time from baseline and diagnostic status. The model included random slope and intercept terms for each participant with age, sex, education and APOE genotype as covariants. The rate of change in NFL for each individual was extracted from the model for subsequent analyses. Associations between plasma NFL concentration and other biomarkers at baseline were tested by multiple linear regression models corrected for age, sex, education and APOE genotype. Associations of baseline plasma NFL concentration or rate of change in plasma NFL with longitudinal data on other biomarkers were tested by LMEMs corrected for age, sex, education and APOE genotype. The Bonferroni correction was used for multiple comparisons. All tests used a significance level of P < 0.05. All statistical analyses were performed using the R programming language, version 3.5.1 (R Foundation) with LME analysis performed specifically using the nlme package, version 3.1.
